# PPARγ activation serves as therapeutic strategy against bladder cancer via inhibiting PI3K-Akt signaling pathway

**DOI:** 10.1186/s12885-019-5426-6

**Published:** 2019-03-07

**Authors:** Shidong Lv, Wei Wang, Hongyi Wang, Yongtong Zhu, Chengyong Lei

**Affiliations:** 1 Department of Urology, Nanfang Hospital, Southern Medical University, No.1838 North of Guangzhou Avenue, Guangzhou, 510515 China; 2 Department of Pathology, General Hospital of Southern Theater Command, PLA, Guangzhou, 510010 China; 3 Department of Obstetrics and Gynecology, Center for Reproductive Medicine, Nanfang Hospital, Southern Medical University, Guangzhou, 510515 China

**Keywords:** Bladder cancer, PPARγ, PI3K, Akt, Apoptosis

## Abstract

**Background:**

Heterogeneity in bladder cancer results in variable clinical outcomes, posing challenges for clinical management of this malignancy. Recent studies suggest both tumor suppressive and oncogenic role of PPARγ in bladder cancer. The fuction of PPARγ signaling pathway in modulating carcinogenesis is controversial.

**Methods:**

The expression of PPARγ and association with overall survival were analyzed in patients from two cohorts. The effect of PPARγ activation on cell proliferation, cell cycle, and cell apoptosis were determined with the agonists (rosiglitazone and pioglitazone), the inverse agonist (T0070907), and the antagonist (GW9662) in Umuc-3 and 5637 bladder cancer cells. The correlation of PPARγ activation with PI3K-Akt pathway was evaluated with RNA sequencing data from the TCGA cases and 30 human bladder cancer cell lines. The effect of PPARγ activation on tumor growth was validated with subcutaneous tumor models in vivo. The effect of PPARγ activation on PI3K-Akt signaling transduction was determined with multiple assays including immunohistochemistry, flow cytometry, proteomic array, and western blotting.

**Results:**

We showed that PPARγ was a favorable prognostic factor in patients with bladder cancer. PPARγ activation by rosiglitazone and pioglitazone markedly induced cell cycle G2 arrest and apoptosis in bladder cancer cells, which resulted in inhibition of cell proliferation in vitro and suppression of tumor growth in vivo. The underlying mechanism involved marked inhibition of PI3K-Akt pathway.

**Conclusions:**

This study reported the tumor-suppressive effect of PPARγ agonists in bladder cancer, suggesting that transactivation of PPARγ could be served as a potential strategy for the chemoprevention and therapeutic treatment of bladder cancer.

**Electronic supplementary material:**

The online version of this article (10.1186/s12885-019-5426-6) contains supplementary material, which is available to authorized users.

## Background

Bladder cancer is the ninth most common type of cancer worldwide, which affects nearly 3.4 million people with 430,000 new cases in 2015 [1]. The highest incidence and mortality are found in North America and Europe, but bladder cancer are increasing in some eastern European and developing countries [2]. Though superficial (non-invasive) urothelial carcinoma with a 5-year survival rate of 96% constitutes the major portion (75%) of bladder cancer cases, patients diagnosed with muscle-invasive bladder cancer (MIBC) havevery poor prognosis, with a 5-year survival rate of 35–70%. Heterogeneity in MIBC results in variable clinical outcomes, posing challenges for clinical management of this aggressive malignancy. In recent years, studies are dedicated to discover the intrinsic molecular subtypes of bladder cancer with particular phenotypic characteristics [3–5]. For example, the frequency of FGFR3 alterations in luminal-papillary subtype (35%) suggests that tyrosine kinase inhibitors of FGFR3 may be an effective therapeutic approach [6]. Most recently, a multiplatform analysis of MIBC patients provided insights into mutational profiles with prognostic value and established a framework associating distinct tumor subtypes with clinical options [7]. These studies have expanded our understanding of this malignancy and provided insights for developing personalized therapies. However, experimental data validating the contribution of these molecular signatures to informing clinical trial designs are limited.

Peroxisome Proliferator Activated Receptor Gamma (PPARG) is a member of the nuclear receptor superfamily that functions as a ligand-activated nuclear receptor that regulates glucose and lipid metabolism, inflammation, and cellular growth and differentiation. Accumulating evidence implicates PPARG as a key gene in bladder cancer development; however, observations regarding the functional effect of PPARG alteration on the development of bladder cancer are controversial. It was reported that luminal MIBCs are enriched with activating FGFR3 and ERBB3 mutations and ERBB2 amplifications, and their gene expression profiles are controlled by PPARγ (the protein name of PPARG), suggesting the pro-tumorigenic role in luminal tumors [3, 4, 7, 8]. In addition, overexpression of GATA3 and FOXA1 may cooperate with PPARγ activation to drive trans-differentiation of a basal bladder cancer cells to a luminal phenotype [9]. Higher expression of PPARγ or its activation by agonists promotes bladder cancer cell migration and invasion [10]. Another in vitro study also showed that reducing PPARγactivity through pharmacologic inhibition or genetic ablation inhibited proliferation of PPARγ-activated bladder cancer cells [11]. On the contrary, multiple studies have indicated that PPARγ activation might be also a promising approach to suppress bladder cancer. By activating PPARγ, simvastatin inhibited bladder cancer cell proliferation and induced cell cycle arrest at G1/G0 phase [12]. The PPARγ agonist troglitazone induces autophagy, apoptosis and necroptosis in bladder cancer cells [13]. In aggregate, PPARγ plays a crucial role in driving cell biology and it is emerging as a promising therapeutic target; however, inconsistent conclusions highlight the urgent need to decipher the underlying mechanism for the diverse characters of PPARγ in modulating bladder cancer.

In the present study, we investigated the association of PPARγ expression with the prognosis of patients from bladder cancer cohorts. The functional effects of PPARγ on cell proliferation, cell cycle and apoptosis were determined in human bladder cancer cells with pharmacological activation and inhibition of PPARγ. The inverse correlation between PPARγactivation and PI3K-Akt signaling pathway was identified with bioinformatics analysis on the The Cancer Genome Atlas (TCGA) datasets and 30 bladder cancer cell lines. The molecular findings were further investigated with human bladder cancer cells as well as subcutaneous tumor model in vivo.

## Methods

### Reagents

Primary antibodies detecting PPARγ, Phosphatase and tensin homolog (PTEN), phospho-Akt (Ser473), p21 Waf1/Cip1, phospho-Bad (Ser136), Phospho-FoxO1 (Ser256), Phospho-Bcl2 (Ser70), and HRP-labeled secondary antibodies were purchased from Cell Signaling Technology (MA, USA). Antibodies detecting phospho-Akt (Ser473) and PPARγ in the immunohistochemistry were purchased from LifeSpan (Seattle, WA, USA). The PPARγagonists rosiglitazone and pioglitazone, the inverse-agonist T0070907, and the antagonist GW9662 were purchased from Selleck Chemicals (TX, USA).

### Human bladder cancer tissue chip

Human bladder cancer tissue microarray (TMA) chip (BlaU066Su01) containing normal mucosa (10 cases) and bladder cancer (56 cases) were obtained from Shanghai Biochip Company Ltd. (Shanghai, China). The information including patient survival and tumor stage has been provided in supplementary data (Additional file 1: Table S1). Samples for TMA were collected using 2.0 mm diameter core needles from a spot of tumors with the most representative histology of each surgical specimen. The clinicopathological and follow-up data of patients were prospectively collected at the Shanghai Eastern Hospital from February 2008 to March 2014. The whole images of histopathological and immunostaining slides were acquired with an Aperio VERSA scanning system (Leica Biosystems, IL, USA).

### Cell culture

The human bladder cancer Umuc-3 cells (Catalog # CRL-1749) was obtained from American Type Culture Collection (ATCC, VA, USA) and cultured in in McCoy’s 5a medium supplemented with 10% fetal bovine serum. The human bladder cancer 5637 cells (HTB-9, Catalog # TCHu1) were purchased from the Cell Bank of Shanghai Institutes for Biological Sciences (Shanghai, China) and cultured in RPMI-1640 medium with 10% fetal bovine serum. The cell lines have recently been tested for mycoplasma contamination and authenticated by Short Tandem Repeat (STR) profiling.

### Cell proliferation, cell cycle and apoptosis

Umuc-3 cells and 5637 cells were seeded in multi-well plates and culture for 24 h before treatment. Cells were treated with rosiglitazone, pioglitazone, T0070907, and GW9662 (10 and 20 μM) for 48 to 96 h. Cell Counting Kit-8 (CCK-8) (Sigma, MO, USA) was used for determining the proliferation of cells.

Flow cytometry was applied to analyze the cell cycle and apoptosis. After treatment, the cells were collected by trypsin digestion and fixed with 70% ethanol for 4 h at 4 °C. Then the cells were washed and resuspended with 500 μl of FxCycle PI/RNase Staining Solution (Invitrogen, Shanghai, China) and incubated in dark for 20 min at 37 °C. Cells were analyzed with FACSCalibur flow cytometer (BD Biosciences, CA, USA). Annexin V-FITC Apoptosis Detection Kit (BD Biosciences) was used for the determination of cell apoptosis according to the instruction of manufacture.

### Subcutaneous tumor model

To establish the subcutaneous bladder tumor model, cultured Umuc-3 and 5637 cells were collected after trypsin digestion and suspended with fresh medium and Matrigel Basement Membrane Matrix (BD Biosciences), and then 10^6^ cells in 0 .1ml were subcutaneously injected into the back of BALB/c athymic nude mice (6-week old, male). For each cell type, 14 animals were randomly devided into two groups with randomization schedules based on body weight. Rosiglitazone was intragastrically administered at 100 mg/kg BW twice a week for 4 weeks (*n* = 7). Animals in the control group (n = 7) were treated with the vehicle solvent (carboxymethylcellulose, 0.5% *w*/*v*). Tumor volume was measured with caliper in two perpendicular diameters of the implant and calculated using formula 1/2a × b^2^, whereas the long diameter and b is the short diameter. Tumor volume was measured once every 7 days. Animal were humanely sacrificed with CO_2_ anesthetization at Day 28. The subcutaneous tumors were removed and fixed in 10% neutral buffered formalin for pathological analysis and immunostaining.

### Immunohistochemical (IHC) staining and immunofluorescence staining

Formalin-fixed tissues were embedded in paraffin for the preparation of 5 μm tissue sections. After deparaffinize and rehydrate the sections, the slides were put in citrate buffer (pH = 6.0) and boiled in a microwave so that antigens are retrieved for 20 min at 98 °C. Then tissue sections were incubated with 3% hydrogen peroxide solution in methanol at room temperature for 10 min to block endogenous peroxidase activity. After blocking with 5% normal goat serum, the sections were incubated with primary antibody overnight at 4 °C. Subsequently, the expression of antigens was visualized with SignalStain Boost Detection Reagent (Cell Signaling Technology) and diaminobenzidine (DAB) followed by the counter staining with hematoxylin. The IHC staining score was estimated for each sample with intensity value (negative,0; +, 1; ++, 2; and +++, 3) multiply positive rate value (negative,0; 1–25%, 1; 26–50%, 2; 51–75%, 3; and 76–100%, 4). Semi-quantitative comparision was analyzed according to the immunostaining score in different types of pathologic lesions.

For immunofluorescence staining, the cultured cells on cover slides were fixed with 4% paraformaldehyde for 15 min at room temperature. Then the cells were blocked with 5% normal goat serum and then incubated with primary antibody overnight at 4 °C, followed by Alexa Fluor 488 or 555 labelled secondary antibody (Abcam, Shanghai, China). Nucleus was counterstained with DAPI (Cell Signaling Technology). The slides were observed and photographed under a fluorescence microscope (Leica DMRA, Wetzlar, Germany).

### Western blotting

The whole cell proteins were isolated from bladder cancer cells. Protein sample mixtures were prepared with LDS Sample Buffer and separated by denaturing gel electrophoresis with the NuPAGE gels (Invitrogen). Then proteins were transferred from gel to a nitrocellulose or polyvinylidene fluoride (PVDF) membrane. After blocking with 5% skim milk, the membranes were incubated with primary antibodies. Beta-actin was used for normalization of protein loading. After incubation with HRP-labeled secondary antibody, the membranes were subsequently developed with Femto Chemiluminescent substrate (Thermo, MA, USA).

### Statistical analysis

All data on cell proliferation, cell cycle and apoptosis rate represent at least three independent experiments and are expressed as mean ± standard deviation. Statistical analysis were performed using one-way Analysis of Variance (ANOVA) followed by Bonferroni’s test for multiple comparisons. *P* value < 0.05 was considered that the difference was statistically significant.

## Results

### PPARγ is a favorable prognostic factor in patients with bladder cancer

**Fig. 1:**
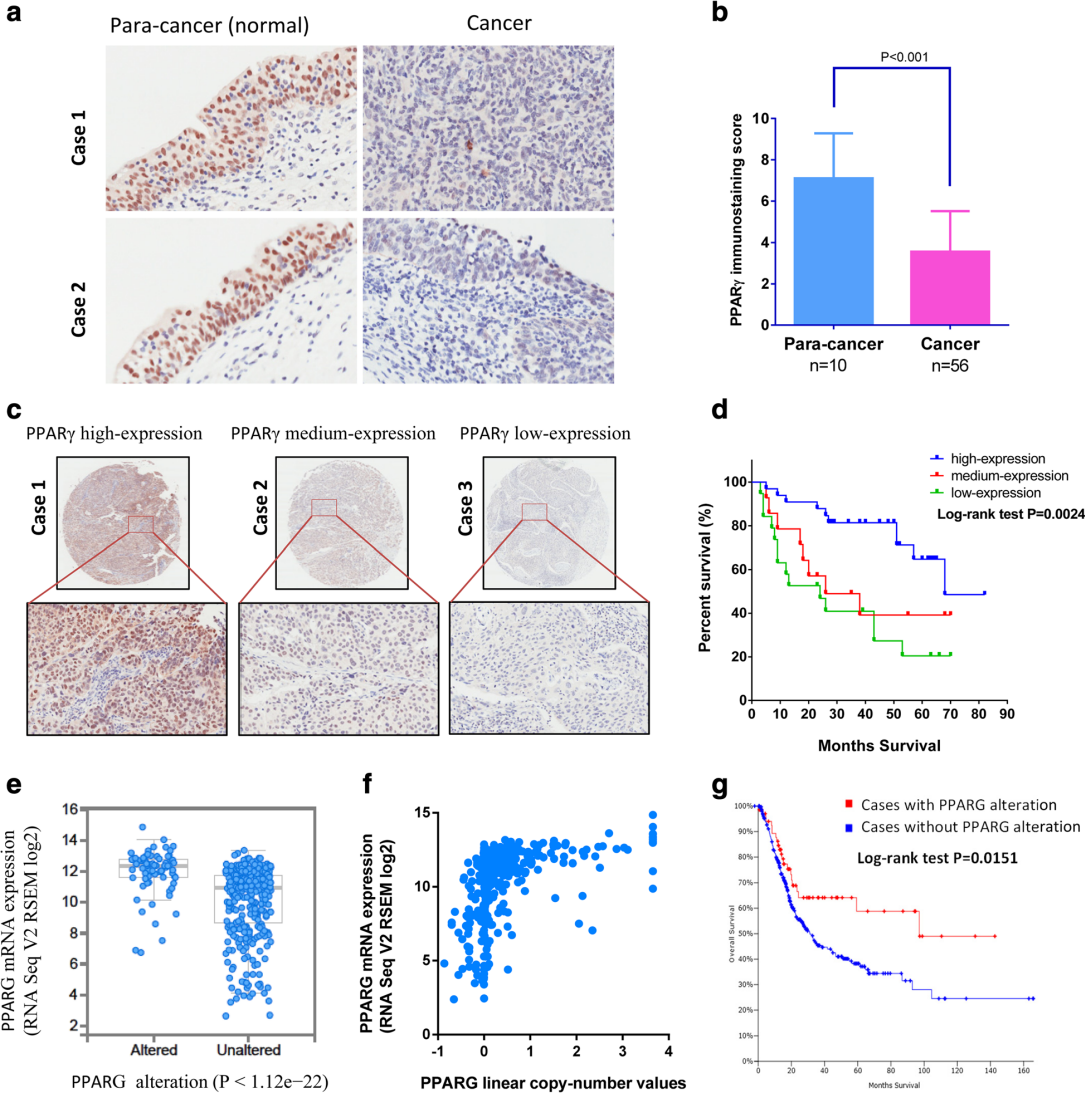
PPARγ is a favorable prognostic factor in patients with bladder cancer. (**a**) Expression of PPARγ in para-cancer (normal) and cancer tissues determined with immunohistochemistry staining. (**b**) PPARγ expression was lower in cancer tissues. (**c**) Different levels of PPARγ expression in bladder cancer cases determined with immunohistochemistry staining. (**d**) Overall survival analysis in bladder cancer cohort (*n* = 66). (**e**) Transcriptional alteration of PPARG mRNA determined by RNA-sequencing in TCGA MIBC cases. (**f**) Correlation of PPARG expression with linear copy-number. (**d**) Overall survival analysis on PPARG alteration in TCGA cohort (*n* = 412)

In the tissue array of a retrospective cohort of 66 patients with bladder cancer, the protein level of PPARγ expression was evaluated by immunohistochemistry staining. PPARγ was highly expressed in para-cancer (normal) tissues, as a nuclear factor predominantly located in the nucleus of cells (Fig. 1a). In contrast, the expression of PPARγ was significantly decreased in the tumor tissues (Fig. 1a and b). The association between PPARγ expression in tumors and the post-surgery overall survival was investigated. The cohort was divided into three groups according to the high-expression (33/66), medium expression (14/66) and low-expression (19/66) of PPARγ (Fig. 1c). Patients survival analysis suggested that high-expression level of PPARγ was associated with longer survival time (*P* = 0.0024) (Fig. 1d).

To confirm this observation, we performed bioinformatics analysis on PPARG with 412 MIBC cases/patients from The Cancer Genome Atlas (TCGA) database. PPARG gene was altered in 86 (21%) of 412 sequenced cases/patients, in which PPARG mRNA expression was dramatically increased (*P* < 1.12e− 22) (Fig. 1e). The overexpression of PPARG was closely associated with the increased copy-number from genome identification of significant targets in cancer (GISTIC) analysis (Fig. 1f). Importantly, the Overall Survival Kaplan-Meier Estimate indicated that MIBC patients with increased mRNA level of PPARG possess significantly longer survival period (*P* = 0.0151) (Fig. 1g), which is in line with the survival analysis on our cohort. In aggregate, these data suggested that PPARγ might be a favorable prognostic factor in bladder cancer patients.

### PPARγ activation suppresses proliferation of bladder cancer cells by inducing G2 phase cell cycle arrest and apoptosis

**Fig. 2:**
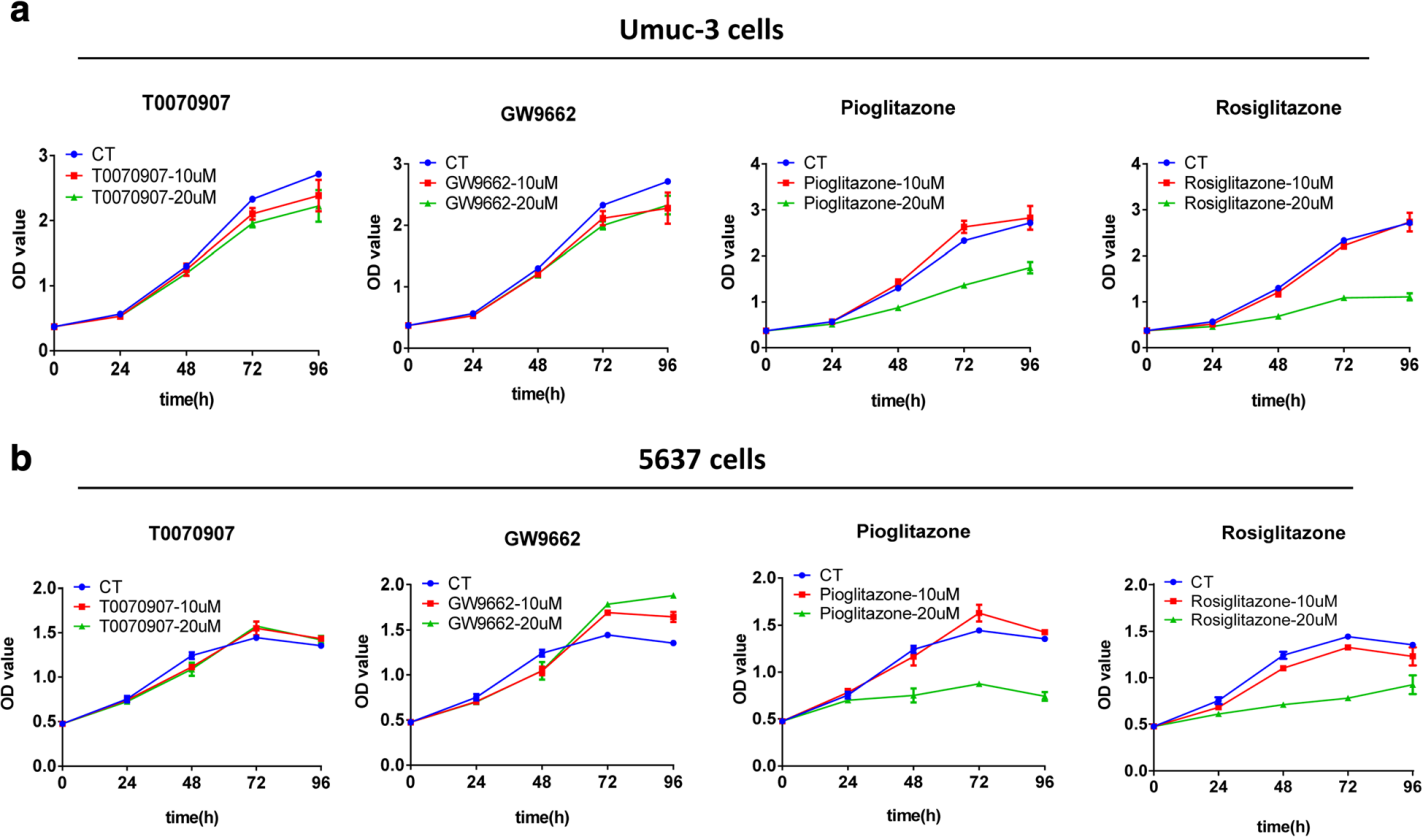
PPARγ agonists inhibited proliferation of bladder cancer cells. (**a**) Cell growth curve for Umuc-3 cells. (**b**) Cell growth curve for 5637 cells. Umuc-3 and 5637 cells were treated with pioglitazone, rosiglitazone, T0070907, and GW9662 (10, 20 μM) for 96 h. Cell proliferation was determined with CCK-8 every 24 h. Data are shown as mean ± standard deviation from triplicate experiments. * signifies *p* < 0.05; **, *p* < 0.01; compared with the control group treated with DMSO (0.1%)

To determine the functional effect of PPARγ in bladder cancer, we next tested the effect of pharmacologic activation and inhibition of PPARγ on the cell growth in Umuc-3 and 5637 cells. In the both cell lines, T0070907, a PPARγinverse-agonist and GW9662, a PPARγantagonist were not able to induce significant inhibition of cell proliferation (Fig. 2a and b). However, the full PPARγagonists including pioglitazone and rosiglitazone significantly suppressed cell growth in Umuc-3 and 5637 cells (Fig. 2a and b), suggesting PPARγ activation suppresses proliferation of bladder cancer cells in vitro.

**Fig. 3:**
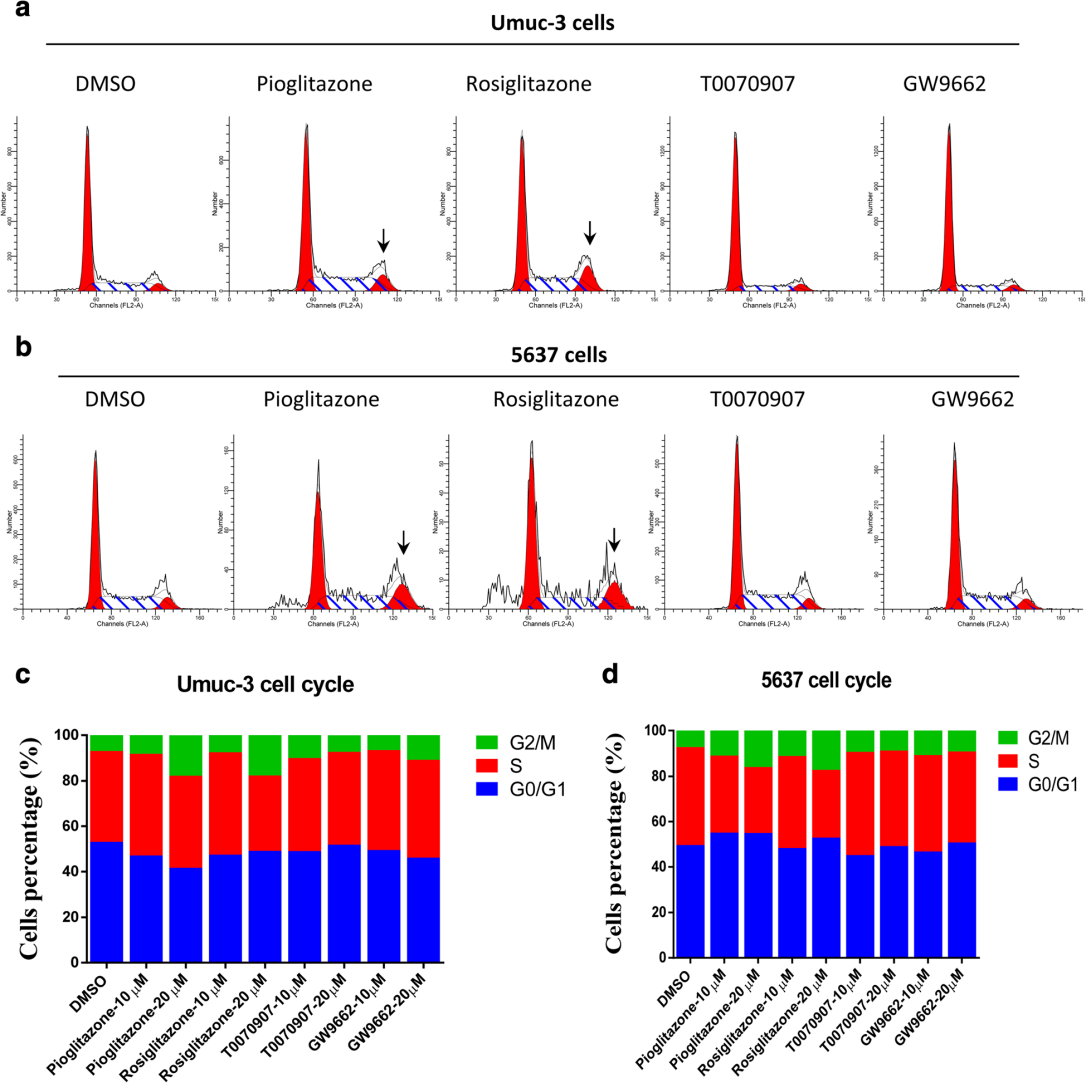
PPARγ agonists induced cell cycle G2 arrest in bladder cancer cells. (**a**) Cell cycle determined with flow cytometry in Umuc-3 cells. (**b**) Cell cycle determined with flow cytometry in 5637 cells. (**c**) Cell cycle distribution Umuc-3 cells. (**d**) Cell cycle distribution in 5637 cells. Umuc-3 and 5637 cells were treated with pioglitazone, rosiglitazone, T0070907, and GW9662 (10, 20 μM) for 72 h

**Fig. 4:**
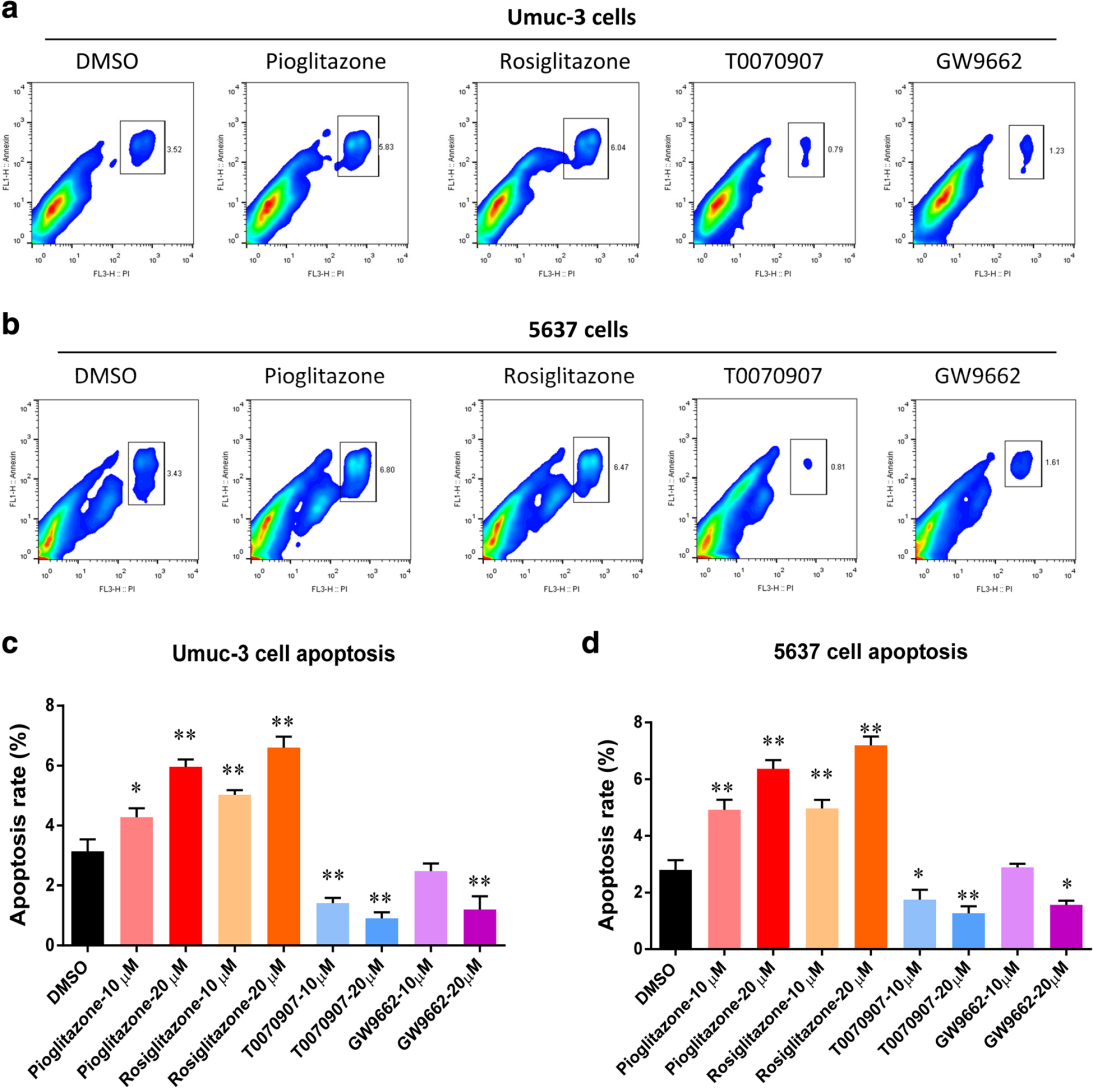
PPARγ agonists induced cell apoptosis in bladder cancer cells. (**a**) Cell apoptosis determined by flow cytometry in Umuc-3 cells. (**b**) Cell apoptosis determined by flow cytometry in 5637 cells. (**c**) Cell apoptosis rate in Umuc-3 cells. (**d**) Cell apoptosis rate in 5637 cells. Umuc-3 and 5637 cells were treated with pioglitazone, rosiglitazone, T0070907, and GW9662 (10, 20 μM) for 72 h. Data are shown as mean ± standard deviation from three independent experiments. * signifies *p* < 0.05; **, *p* < 0.01; compared with the control group treated with DMSO (0.1%)

Next we determined the effect of PPARγ activation or inhibition on the cell cycle progression of bladder cancer cells. In Umuc-3 and 5637 cells, pioglitazone and rosiglitazone markedly increased the proportion of G2 phase cells, however, cell population at G1 phase was not significantly altered (Fig. 3). The inhibition of PPARγ by T0070907 and GW9662 did not induce significant change on cell cycle progression in these cells. In addition, PPARγ activation by pioglitazone and rosiglitazone dramatically increased the apoptosis in Umuc-3 and 5637 cells (Fig. 4). In contrast, T0070907 and GW9662 induced significant suppression of apoptosis in the bladder cancer cells. Taken together, these data indicated that PPARγ activation suppresses proliferation of bladder cancer cells by inducing G2 phase cell cycle arrest and apoptosis.

### PPARγ activation inhibits phosphoinositide 3-kinase (PI3K) -Akt signaling pathway in bladder cancer

**Fig. 5:**
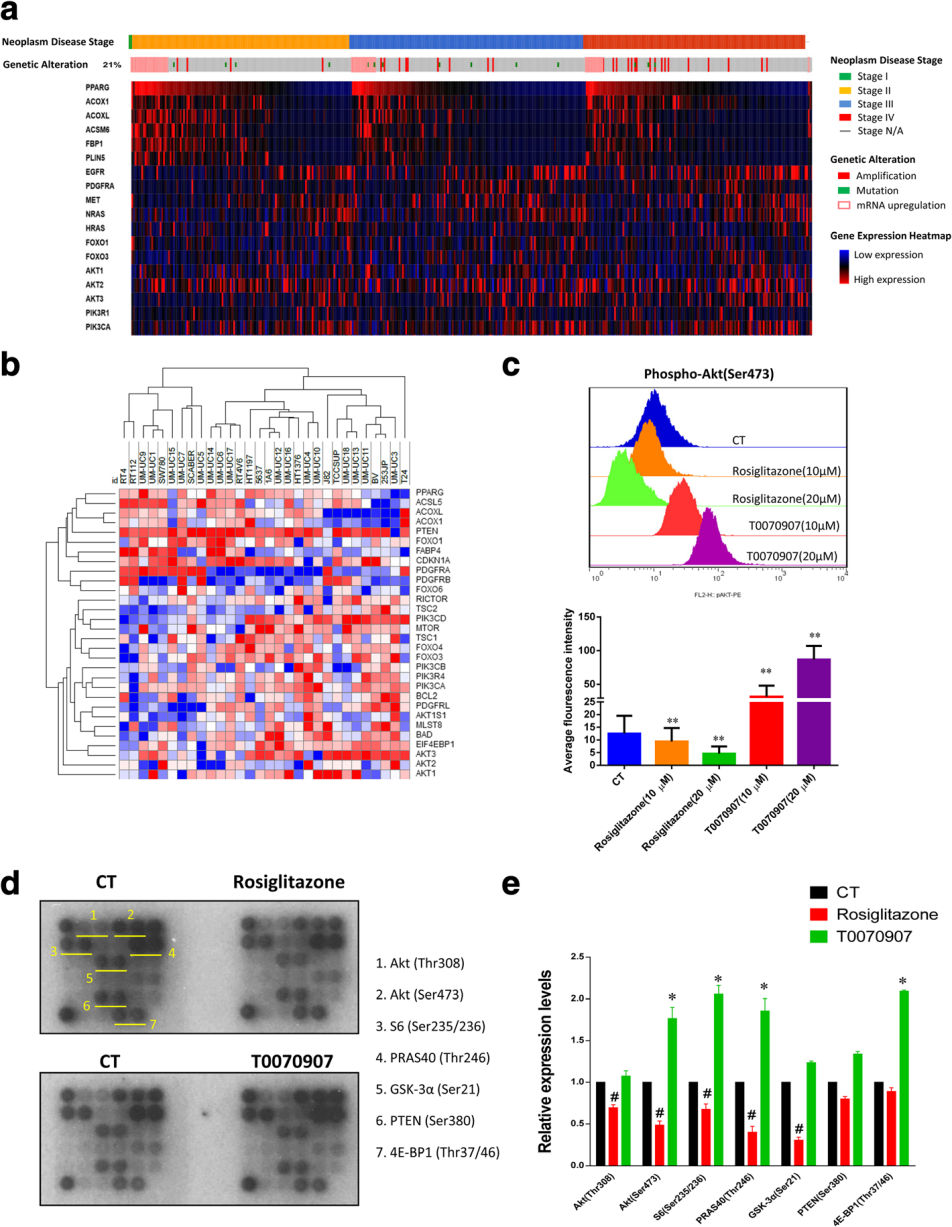
PPARγ activation inhibits PI3K-Akt signaling pathway in bladder cancer. (**a**) Heat Map of PPARG and PI3K-Akt associated genes in TCGA cases. (**b**) Hierarchical Clustering of PPARG and PI3K-Akt associated genes in 30 human bladder cancer cell lines. (**c**) Phosphorylation of Akt (Ser473) determined with flow cytometry. 5637 cells were treated with rosiglitazone and T0070907 (10, 20 μM) for 12 h. * signifies *p* < 0.05; **, *p* < 0.01; compared with the control group treated with DMSO (0.1%). (d) Proteomic array analysis on Akt signaling pathway. 5637 cells were treated with rosiglitazone and T0070907 (20 μM) for 72 h. (**e**) Quantitative analysis of proteomic array. Data are shown as mean ± standard deviation. #, *p* < 0.05 decreased when compared with the control (CT); *, *p* < 0.05 increased when compared with the control

We next analyzed the transcriptional impact of PPARG in the TCGA MIBC cohort by stratification into three major groups according to Neoplasm Disease Stage. We found that PPARG mRNA expression was in parallel with PPARγ activation as indicated by the expression of its typical target genes including ACOX1, ACOXL, ACSM6, FBP1 and PLIN5. Interestingly, the expression of signature genes of PI3K-Akt signaling pathway was commonly up-regulated in the MIBC patients with low activation of PPARγfrom different stages of bladder cancer (Fig. 5a). The inverse association was also observed from the RNA sequencing analysis with 30 human bladder cancer cell lines (Fig. 5b). These data suggests that the interaction of PPARγwith PI3K-Akt pathway may drive the pathogenesis of bladder cancer.

To decipher the correlation between PPARγand PI3K-Akt, we determined the effects of PPARγ selective agonist (rosiglitazone) and the inverse-agonist (T0070907) on Akt phosphorylation/activation in 5637 cells. As shown in Fig. 5c, rosiglitazone induced significant inhibition of Akt phosphorylation (Ser473). In contrast, T0070907 dramatically promoted the activation of Akt. In addition, we further validated the effect of rosiglitazone and T0070907 on PI3K-Akt signaling activation with a protein array (Fig. 5d). In consistent with the Phos-flow assay, phosphorylation of Akt (Thr308 and Ser473) and its down-stream molecules including S6, PRAS40 and GSK-3α was significantly repressed by rosiglitazone, however, enhance by T0070907 (Fig. 5e). Together, these data suggests that PPARγ activation inhibits PI3K-Akt signaling pathway in bladder cancer cells.

### PPARγ activation suppresses bladder cancer through inhibiting Akt pathway

**Fig. 6:**
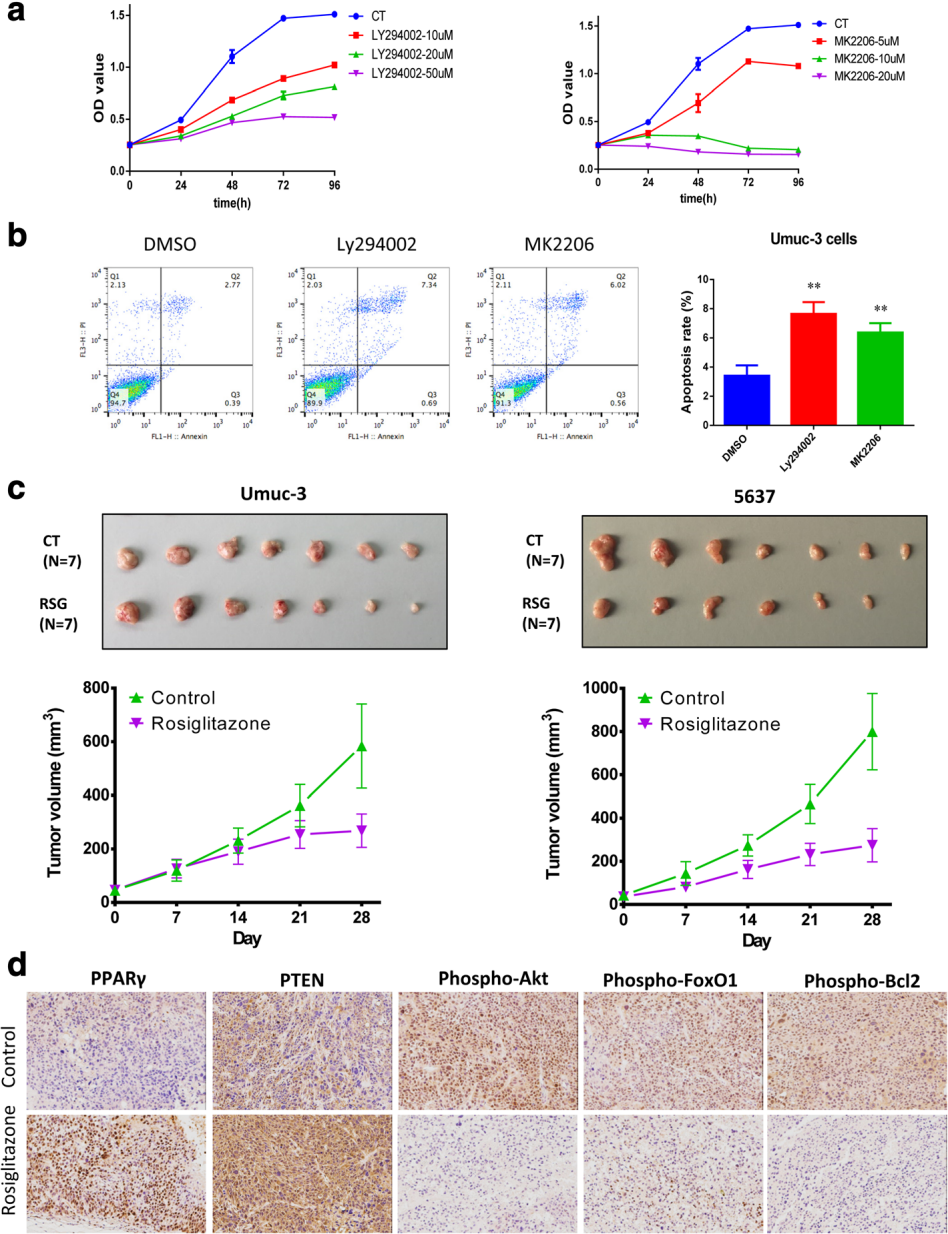
PPARγ activation suppresses bladder cancer through inhibiting Akt pathway. (**a**) Blockade of PI3K-Akt signaling pathway inhibited proliferation of bladder cancer cells. Umuc-3 cells were treated with Ly294002 (10, 20, 50 μM) and MK2206 (5, 10, 20 μM) for 96 h. Cell proliferation was determined with CCK-8 every 24 h. (**b**) Blockade of PI3K-Akt signaling pathway induced cell apoptosis in bladder cancer cells. Umuc-3 cells were treated with Ly294002 (20 μM) and MK2206 (10 μM) for 24 h. Data are shown as mean ± standard deviation from triplicate experiments. * signifies *p* < 0.05; **, *p* < 0.01; compared with the control group treated with DMSO (0.1%). (**c**) Rosiglitazone hindered the growth of subcutaneous tumors generated with Umuc-3 and 5637 cells in nude mice (*n* = 7). (**d**) IHC staining of PPARγ, PTEN, and the phosphorylation of Akt (Ser473), FoxO1 (Ser256) and Bcl2 (Ser70) in subcutaneous tumors

To bridge the modulation of PPARγon PI3K-Akt pathway with its functional effects on the growth of bladder cancer cells, we next determined the Akt-modulated molecules that govern cell survival and apoptosis. Firstly, we validated that blockade of PI3K-Akt signaling pathway using the specific inhibitor of PI3K, Ly294002 and the highly selective inhibitor of Akt, MK2206 repressed proliferation of Umuc-3 cells in a dose-dependent manner (Fig. 6a). Also, Ly294002 and MK2206 induced remarkable apoptosis in the Umuc-3 cells (Fig. 6b), suggesting PI3K-Akt plays crucial role in growth control of bladder cancer cells.

In further, we confirmed the suppressive effect of PPARγ activation on Akt phosphorylation in bladder cancer with the subcutaneous tumor models. In parallel with the results from in vitro studies, treatment with rosiglitazone significantly hindered the growth of tumors that subcutaneously generated with Umuc-3 and 5637 cells (Fig. 6c). Importantly, we observed that PPARγ activation in the tumor tissues was in line with the expression of the target gene PTEN, but inversely correlated with the activation of Akt, as indicated by the phosphorylation of Akt (Ser473) and its downstream FoxO1 (Ser256) and Bcl2 (Ser70) (Fig. 6d).

**Fig. 7:**
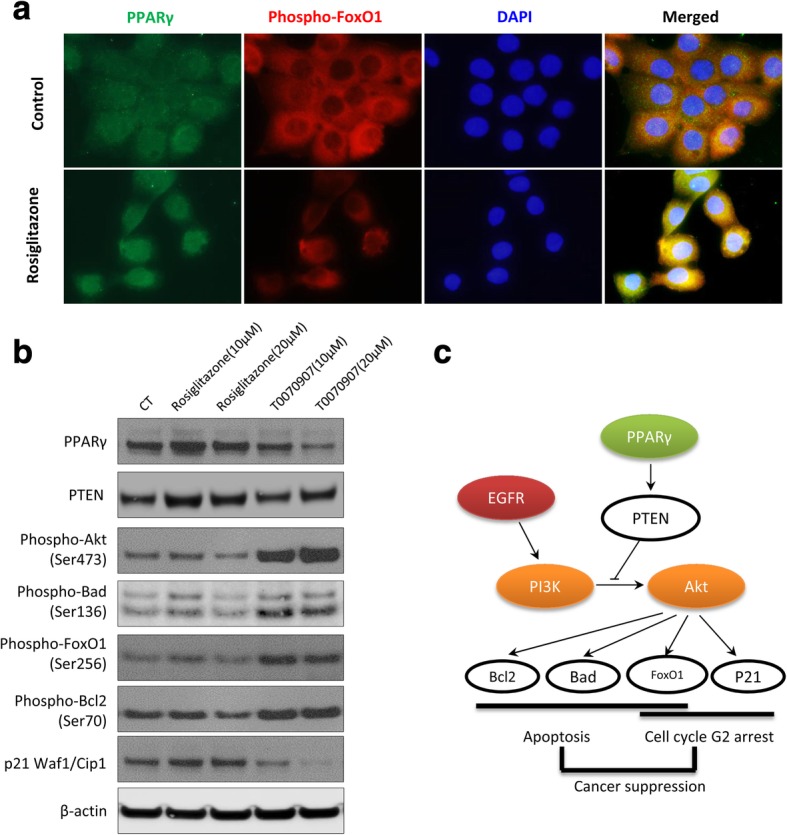
PPARγ activation suppresses signaling transduction that controls cell cycle and apoptosis in Akt pathway. (**a**) Immunofluorescence staining of PPARγ and phospho-FoxO1 (Ser256) in 5637 cells. Cells seeded on cover slides were treated with solvent (0.1%DMSO) or rosiglitazone (20 μM) for 12 h. (**b**) Western blotting of PPARγ and downstream molecules in Akt pathway. 5637 cells were treated with rosiglitazone and T0070907 (10, 20 μM) for 72 h. (**c**) Schematic illustration of the suppressive effect of PPARγ activation against bladder cancer

In addition, the immunofluorescence staining showed that rosiglitazone induced translocation of PPARγ from cytoplasm into nucleus indicating marked PPARγ activation in 5637 cancer cells (Fig. 7a). Simultaneously, the phosphorylation of forkhead transcription factor FoxO1 (Ser256) was significantly decreased, which resulted the cytoplasmic sequestration of FoxO1 and inactivated function of promoting cell cycle arrest and apoptosis. Finally, we further validated the association of PPARγ with the Akt-regulated downstream molecules that governing cell survival and apoptosis. Treatment with rosiglitazone significantly increased expression of PPARγ and PTEN. In contrast, down-regulation of PPARγ by T0070907 induced marked activation of Akt and the subsequent phosphorylation of Bad, FoxO1, and Bcl2; however, negatively regulated the cyclin dependent kinase inhibitor p21 Waf1/Cip1 (Fig. 7b). In aggregate, these data suggests that the inhibition of Akt signaling pathway might serve as an important mechanism underlying the suppression of bladder cancer by PPARγ activation.

## Discussion

Recent genomic analysis suggested potential risk of bladder cancer upon activation of PPARγ signaling pathway. PPARG was identified as a significant focal amplification specifically in bladder cancer by genome identification of significant targets in cancer (GISTIC) analysis [14]. The amplification and overexpression of PPARG occurred in about 40% MIBC cases [15]. Accumulating evidence implicates PPARG as a key gene in bladder cancer, but the functional effect of PPARG in tumor development are controversial. The pro-tumorigenic and anti-tumorigenic role of PPARG was also found in colon cancer, breast cancer, prostate cancer, lung cancer and many others [16]. In this study, we found MIBC patients from our cohort and the TCGA database markedly benefited from higher expression levels of PPARG with longer overall survival, suggesting the favorable action of PPARγ in bladder cancer. In MIBC cohort and human bladder cancer cell lines, the expression of PPARG and its target genes were found inversely associated with the activation of PI3K-Akt pathway. Further experiments confirmed that pharmacological activation of PPARγ notably induced cell cycle arrest and apoptosis of bladder cancer cells, which was tightly related to the inhibition of signaling transduction in PI3K-Akt pathway. Our data suggested that PPARγ activation suppressed bladder cancer through inhibiting PI3K-Akt signaling pathway (Fig. 7c).

PPARγis an important modulator of cellular energy metabolism that has been implicated in urothelial differentiation and malignancy. Rosiglitazone and other synthetic ligands of PPARγ have been used as insulin sensitizers for the treatment of type 2 diabetes. However, undesirable side effects including weight gain, fluid retention, bone loss, and congestive heart failure have limited the use of thiazolidinediones. More importantly, the PPARγ agonist pioglitazone was shown to be associated with an increased risk of bladder cancer [17]. It was hypothesized that the effect of pioglitazone on promoting bladder cancer might be due to its PPARα activity [18], as this was not observed with rosiglitazone, which is highly selective for PPARγ [19]. The results of large population based studies indicated that pioglitazone was associated with an increased risk of bladder cancer, but the absence of an association with rosiglitazone suggested that the increased risk was drug specific and not a class effect [20]. In contrast, other population-based studies indicated that pioglitazone use was not associated with an increased risk of bladder cancer [21], or there was even trends of decreased bladder cancer [22]. On the other hand, another new class of PPARγ specific agonists, PPARγ-active DIM-Cs significantly inhibited bladder tumorigenesis [23]. In line with previous studies, our data also suggested that PPARγ activation by agonists significantly suppressed bladder tumor growth in vitro and in vivo.

The regulation of PI3K by PPARγ from previous studies are also controversial. It was reported that rosiglitazone can activate PI3K-Akt through PPARγ-dependent biosynthesis of VEGF and leptin [24, 25]. On the contrary, PPARγ agonists were found to upregulate PTEN, subsequently inhibiting the PI3K-Akt signaling pathway in lung cancer cells [26]. Previous study also indicated that the tumor suppressor and anti-inflammatory actions of PPARγ agonists were mediated via upregulation of PTEN in Caco2 colorectal cancer cells and MCF7 breast cancer cells [27]. PTEN has been identified as lost or mutated in several sporadic and heritable cancer types. Experimental studies have indicated that PTEN is a non-redundant phosphatase that is essential for regulating the highly oncogenic pro-survival PI3K-Akt signaling pathway [28]. In bladder cancer, PI3K pathway alterations are commonly observed [29], and the inactivation of PTEN has also been implicated in the development of bladder cancer [30]. For other cancer types, some small molecule agonist of PPARγ has been shown to reduce the phosphorylation of Akt at Ser473 and exert therapeutic effects [31, 32]. In this study, we also observed the negative correlation between the expression of PTEN and Akt activation under the treatment of PPARγ agonists. However, in addition to PPARγ, numerous factors have been demonstrated to upregulate PTEN transcription including IGF-2, EGR-1, and p53 [28]. Therefore, as a limitation in this study, it was difficult to conclude whether the anti-cancer effect of PPARγ activation was dependent on PTEN without gene-knockout validations.

In addition, the transactivation of PPARG involves interaction with coactivators such as retinoid X receptors (RXRs), and the transrepression involves interference with other signal transduction pathways including NFκB, STAT, and AP1 [33]. The crosstalk of PPARγ signaling transduction with these factors determines the fate of cellular pathogenesis. From the view of tumor microenvironment, other biological functions of PPARγmay be also involved in bladder cancer. For example, a recent study characterized diverse genetic alterations in MIBC that convergently lead to constitutive activation of PPARγ/RXRα and result in immunosurveillance escape by inhibiting CD8+ T-cell recruitment [15].Thus, the outcome of PPARγ agonists use might be variable in different circumstances of tumorigenesis including cancer types and disease stages. In the present study, we focused on the intrinsic changes of bladder cancer cells that induced by the alteration of PPARγ. Based on the analysis of PI3K-Akt pathway in TCGA cohort and 30 human bladder cell lines (Fig. 5a and b), a group of genes governing cell survival and apoptosis were identified inversely correlated with PPARγ transactivation. We further validated in bladder cancer Umuc-3 and 5637 cells that phosphorylation of Bcl2, Bad, and FoxO1 was consistently repressed by PPARγ agonist rosiglitazone, but enhanced by the inverse agonist T0070907. The basal cellular transactivation of PPARG may affect the responses of cells to PPARγ agonists, but our data suggested that the cell lines with low and medium basal levels of PPARG expression were both sensitive to the PPARγ agonists. Similarly, other investigators also found that PPARγ antagonist GW9662 recovered the cell cycle arrest induced by simvastatin in T24, 5637, and EJ cells [12]. Another high-affinity ligand for PPARγ, ciglitazone, induced G2/M cell cycle arrest characterized by an overexpression of p53, p21 and p27 in RT4 cells, and also triggered apoptosis in T24 cells [34]; however, the cell cycle arrest and induction of apoptosis was proposed through up-regulation of TRAIL that was independent of PPARγ activation [35]. Taken together, though experimental studies showed promising anti-malignant effect of PPARγ agonists, due to the heterogeneity of this malignancy, more precise characterization on both genetic and epigenetic alterations in patients as well as cell lines is warranted to better understand the multifacet role of PPARγ in bladder cancer.

## Conclusions

This study demonstrated the tumor-suppressive effect of PPARγ agonists rosiglitazone and pioglitazone in bladder cancer. We showed that PPARγ was a favorable prognostic factor in patients with bladder cancer. PPARγ activation by rosiglitazone and pioglitazone markedly induced cell cycle G2 arrest and apoptosis in bladder cancer cells, which resulted in inhibition of cell proliferation in vitro and suppression of tumor growth in vivo. The underlying mechanism involved inhibition of PI3K-Akt pathway. This study suggested that transactivation of PPARγ could be served as a potential strategy for the chemoprevention and therapeutic treatment of bladder cancer.

## Additional file

Additional file 1: **Table S1.** The information of Human bladder cancer cases used in tissue microarray (TMA). (PDF 59 kb)

## Abbreviations

ANOVA: one-way analysis of variance
ATCC: American type culture collection
CCK-8: Cell counting Kit-8
FGFR3: Fibroblast growth factor receptor 3
GISTIC: genome identification of significant targets in cancer
IHC: Immunohistochemistry
MIBC: muscle-invasive bladder cancer
PI: propidium iodide
PI3K: Phosphoinositide 3-kinase
PPARγ: Peroxisome proliferator-activated receptor gamma
PTEN: Phosphatase and tensin homolog
TCGA: The cancer genome atlas
TMA: tissue microarray
