# Supplementary material for: PPARγ activation serves as therapeutic strategy against bladder cancer via inhibiting PI3K-Akt signaling pathway
Source: BMC Cancer. 2019 Mar 7;19:204. doi: 10.1186/s12885-019-5426-6 (PMC6407222; doi:10.1186/s12885-019-5426-6)
Supplement: Supplementary file 1 — Table S1. The information of Human bladder cancer cases used in tissue microarray (TMA). (PDF 59 kb) [file 12885_2019_5426_MOESM1_ESM.pdf]

**Supplementary table.** The information of Human bladder cancer cases used in tissue microarray (TMA)

| Case No. | Tissue code | Age range (year) | Survival time range (year) | Survial state | Tumor size | TNM Stage | | | AJCC Stage |
|---|---|---|---|---|---|---|---|---|---|
| 1 | RUrBla0706A0036 | 70-80 | 5.0-6.0 | Live | 7*4*2.5cm | T2 | N/A | M0 | N/A |
| 2 | RUrBla0706A0037 | 80-90 | 6.0-7.0 | Live | 6.5*6.5*1.5cm | T3 | N0 | M0 | 3 |
| 3 | RUrBla0706A0038 | 60-70 | 2.0-3.0 | Dead | 2.5*2*0.2cm | T2 | N0 | M0 | 2 |
| 4 | RUrBla0706A0039 | 60-70 | 2.0-3.0 | Dead | 4*3.5*1.5cm | T2 | N0 | M0 | 4 |
| 5 | RUrBla0706A0040 | 50-60 | 0.5-1.0 | Dead | 5*2.5*1.5cm | T2 | N0 | M0 | 2 |
| 6 | RUrBla0710A0043 | 60-70 | 1.0-2.0 | Dead | 3*3*1cm | T1 | N0 | M0 | 1 |
| 7 | RUrBla0710A0044 | 40-50 | 0-0.5 | Dead | 4.5*4*1.5cm | T2 | N0 | M0 | 4 |
| 8 | RUrBla0710A0045 | 50-60 | 0.5-1.0 | Dead | 3*2*1cm, 1*1*0.5cm | T1 | N/A | M0 | N/A |
| 9 | RUrBla0711A0051 | 80-90 | 5.0-6.0 | Live | 9*7*2cm | Tis | N0 | M0 | 0is |
| 10 | RUrBla0712A0058 | 40-50 | 0-0.5 | Dead | 7*6*6cm | T3 | N/A | M0 | N/A |
| 11 | RUrBla0712A0059 | 70-80 | 4.0-5.0 | Live | 2*1*0.9cm | T2 | N0 | M0 | 2 |
| 12 | RUrBla0712A0060 | 80-90 | 5.0-6.0 | Dead | 2*2*1cm, 1.5*1.5*1cm | T1 | N0 | M0 | 1 |
| 13 | RUrBla0712A0061 | 60-70 | 1.0-2.0 | Dead | 2*2*1cm | T3 | N0 | M0 | 3 |
| 14 | RUrBla0803A0066 | 70-80 | 4.0-5.0 | Dead | 6*6*1cm | T4a | N0 | M0 | 4 |
| 15 | RUrBla0804A0068 | 60-70 | 1.0-2.0 | Dead | 1.5*1*0.5cm, 2*2*2cm | T1 | N/A | M0 | N/A |
| 16 | RUrBla0806A0071 | 80-90 | 5.0-6.0 | Live | 5*4*2.5cm | T2 | N0 | M0 | 2 |
| 17 | RUrBla0807A0074 | 60-70 | 1.0-2.0 | Dead | 2*1.5*0.5cm | T3 | N0 | M0 | 3 |
| 18 | RUrBla0807A0075 | 70-80 | 4.0-5.0 | Live | 2*1.8*1cm | T3 | N0 | M0 | 3 |
| 19 | RUrBla0807A0076 | 50-60 | 0.5-1.0 | Dead | 5*4.8*3cm | T3 | N/A | M0 | N/A |
| 20 | RUrBla0808A0078 | 80-90 | 5.0-6.0 | Live | 3*2*1cm | T2 | N0 | M0 | 2 |
| 21 | RUrBla0808A0079 | 70-80 | 4.0-5.0 | Live | 5*3*1cm | T1 | N0 | M0 | 1 |
| 22 | RUrBla0811A0083 | 70-80 | 5.0-6.0 | Live | 4*3.5*2.5cm | T2 | N0 | M0 | 2 |
| 23 | RUrBla0812A0084 | 70-80 | 5.0-6.0 | Live | 5*4.5*3cm | T2 | N0 | M0 | 2 |
| 24 | RUrBla0901A0088 | 70-80 | 5.0-6.0 | Live | 4*2*1.5cm | T3 | N/A | M0 | N/A |
| 25 | RUrBla0901A0090 | 50-60 | 0.5-1.0 | Dead | 2*2*2cm, 3*3*2.5cm | T3 | N0 | M0 | 4 |
| 26 | RUrBla0901A0091 | 70-80 | 5.0-6.0 | Live | 5*3*3cm | T3 | N0 | M0 | 3 |
| 27 | RUrBla0902A0095 | 70-80 | 5.0-6.0 | Live | 6*6*5.5cm | T2 | N/A | M0 | N/A |
| 28 | RUrBla0904A0097 | 70-80 | 3.0-4.0 | Live | 7*6.5*1cm | Tis | N0 | M0 | 0is |
| 29 | RUrBla0905A0102 | 60-70 | 0.5-1.0 | Dead | 1.7*1.5*1cm | T1 | N/A | M0 | N/A |
| 30 | RUrBla0906A0103 | 70-80 | 4.0-5.0 | Dead | 5*3.5*2cm | T1 | N0 | M0 | 1 |
| 31 | F03A0105 | 50-60 | 0.5-1.0 | Dead | 5*5*3cm | T3 | N0 | M0 | 3 |
| 32 | F03A0108 | 70-80 | 4.0-5.0 | Live | 4*3.5*3.5cm | T3 | N0 | M0 | 3 |
| 33 | F03A0110 | 50-60 | 0-0.5 | Dead | 4*3*1.5cm | T3 | N0 | M0 | 3 |
| 34 | F03A0112 | 50-60 | 0-0.5 | Dead | 3*3*1.5cm | T4a | N/A | M0 | 4 |

| | | | | | | | | | |
|---|---|---|---|---|---|---|---|---|---|
| 35 | F03A0120 | 70-80 | 3.0-4.0 | Dead | 5*5*1.5cm | T3 | N0 | M0 | 3 |
| 36 | F03A0121 | 70-80 | 4.0-5.0 | Live | 4*3.5*1cm | T3 | N0 | M0 | 3 |
| 37 | F03A0122 | 70-80 | 2.0-3.0 | Live | 3*2.5*0.9cm | T3 | N0 | M0 | 3 |
| 38 | F03A0123 | 70-80 | 4.0-5.0 | Live | 2*2*1cm | T3 | N0 | M0 | 3 |
| 39 | F03A0124 | 40-50 | 0-0.5 | Dead | 6*5.5*1.5cm | T3 | N0 | M0 | 4 |
| 40 | F03A0126 | 60-70 | 2.0-3.0 | Dead | N/A | N/A | N/A | M0 | N/A |
| 41 | F03A0137 | 70-80 | 2.0-3.0 | Live | 2.5*2.5*1cm | Tis | N/A | M0 | N/A |
| 42 | F03A0127 | 70-80 | 4.0-5.0 | Live | N/A | N/A | N/A | M0 | N/A |
| 43 | F03A0131 | 60-70 | 1.0-2.0 | Dead | 3*2*2cm | T3 | N/A | M0 | N/A |
| 44 | F03A0133 | 70-80 | 3.0-4.0 | Live | 8*5*2.5cm | T1 | N0 | M0 | 1 |
| 45 | F03A0134 | 70-80 | 3.0-4.0 | Dead | 7*6*6cm | T1 | N0 | M0 | 1 |
| 46 | F03A0141 | 60-70 | 2.0-3.0 | Live | 4.5*2.5*1cm | Tis | N0 | M0 | 0is |
| 47 | F03A0216 | 60-70 | 1.0-2.0 | Dead | 4*3*2cm | T3 | N0 | M0 | 3 |
| 48 | F03A0218 | 70-80 | 3.0-4.0 | Live | 3.5*2.5*1.5cm | T2 | N0 | M0 | 4 |
| 49 | F03A0219 | 70-80 | 3.0-4.0 | Live | 3*2*1.5cm | Tis | N/A | M0 | N/A |
| 50 | F03A0220 | 70-80 | 3.0-4.0 | Live | 4*3*1.5cm | T2 | N0 | M0 | 2 |
| 51 | F03A0221 | 60-70 | 1.0-2.0 | Live | 3.5*3*1.5cm | T3 | N0 | M0 | 3 |
| 52 | F03A0222 | 50-60 | 0.5-1.0 | Dead | 3*3*1.5cm | T3 | N0 | M0 | 3 |
| 53 | F03A0224 | 60-70 | 1.0-2.0 | Live | 1.5*1.5*1cm, 2*2*1cm | T1 | N/A | M0 | N/A |
| 54 | F03A0230 | 60-70 | 1.0-2.0 | Live | 3*2*1cm | T2 | N0 | M0 | 2 |
| 55 | F03A0231 | 50-60 | 0.5-1.0 | Dead | 3*2.5*1.5cm | T3 | N0 | M0 | 3 |
| 56 | F03A0347 | 60-70 | 2.0-3.0 | Live | 6*5*3cm | T2 | N0 | M0 | 2 |

Note: TNM, TNM Classification of Malignant Tumors; AJCC, American Joint Committee on Cancer; N/A, not applicable or unknown.
